# The clinical and molecular epidemiology of *Staphylococcus aureus* infections in Fiji

**DOI:** 10.1186/1471-2334-14-160

**Published:** 2014-03-24

**Authors:** Adam Jenney, Deborah Holt, Roselyn Ritika, Paul Southwell, Shalini Pravin, Eka Buadromo, Jonathan Carapetis, Steven Tong, Andrew Steer

**Affiliations:** 1 Fiji Group A Streptococcal Project, University of Melbourne, Victoria, Australia; 2 Centre for International Child Health, University of Melbourne, Flemington Road, Victoria 3052, Parkville, Australia; 3 Menzies School of Health Research, Northern Territory, Darwin, Australia; 4 Microbiology Department, Royal Darwin Hospital, Northern Territory, Suva, Fiji; 5 Microbiology Department, Colonial War Memorial Hospital, Suva, Fiji; 6 Telethon Institute for Child Health Research, Centre for Child Health Research, University of Western Australia, Western Australia, Perth, Australia; 7 Group A Streptococcal Research Group, Murdoch Childrens Research Institute, Melbourne, Australia

**Keywords:** *Staphylococcus aureus*, Clonal complex, Typing, Antimicrobial susceptibility

## Abstract

**Background:**

There are few data describing the microbiology and genetic typing of *Staphylococcus aureus* that cause infections in developing countries.

**Methods:**

In this study we observed *S. aureus* infections in Pacific Island nation of Fiji in both the community and hospital setting with an emphasis on clonal complex (CC) genotyping and antimicrobial susceptibility.

**Results:**

*S. aureus* was commonly found in impetigo lesions of school children and was recovered from 57% of impetigo lesions frequently in conjunction with group A streptococcal infection. Methicillin-resistant *S. aureus* (MRSA) comprised 7% (20/299) of isolates and were all non-multi-resistant and all genotyped as CC1. In contrast, there was a diverse selection of 17 CCs among the 105 genotyped methicillin-susceptible *S.aureus* (MSSA) strains. Isolates of the rare, phylogenetically divergent and non-pigmented CC75 lineage (also called *S.argenteus*) were found in Fiji.

From hospitalized patients the available 36 MRSA isolates from a 9-month period were represented by five CCs. The most common CCs were CC1 and CC239. CC1 is likely to be a community-acquired strain, reflecting what was found in the school children, whereas the CC239 is the very successful multi-drug resistant MRSA nosocomial lineage. Of 17 MSSA isolates, 59% carried genes for Panton-Valentine leukocidin. The *S. aureus* bacteraemia incidence rate of 50 per 100,000 population is among the highest reported in the literature and likely reflects the high overall burden of staphylococcal infections in this population.

**Conclusions:**

*S. aureus* is an important cause of disease in Fiji and there is considerable genotypic diversity in community skin infections in Fijian schoolchildren. Community acquired- (CA)- MRSA is present at a relatively low prevalence (6.7%) and was solely to CC1 (CA-MRSA). The globally successful CC239 is also a significant pathogen in Fiji.

## Background

*Staphylococcus aureus* is one of the most successful human pathogens. The dispersion of certain successful lineages can be tracked across the globe [1]. Since first being recognised in the early 1960s, methicillin-resistant *S. aureus* (MRSA) emerged as an important worldwide pathogen associated with nosocomial infections in the developed world, so-called healthcare-associated MRSA (HA-MRSA) [2]. In the last two decades however, community-associated MRSA (CA-MRSA) strains have been observed to cause disease in people without healthcare contact. At-risk groups have been identified, including sports teams, prisoners, military personnel, and Indigenous population groups. In addition, *S. aureus* lineages have demonstrated a degree of geographical restriction [3].

While extensive data on the burden of disease due to *S. aureus* are available from many industrialised countries, there are few data from developing countries. In an analysis of 220 papers concerning hospital-acquired infection in developing countries, *S. aureus* was the second most recognised cause after the Enterobacteriaceae; antibiotic resistance was recorded in just eight of the studies reviewed and, of these, 54% of the *S.aureus* isolates were found to be methicillin resistant [4]. In a study of *S. aureus* from rural Thailand, *S.aureus* caused similar clinical manifestations of disease as those seen in more developed countries but an increased mortality was noted [5]. Within developed countries, *S. aureus* has been found to cause significant disease amongst certain disadvantaged populations, including Indigenous communities [6]. For example, in a study of impetigo in an urbanised population of Indigenous Australians in Far North Queensland in Australia, *S. aureus* was isolated from 92 of 118 cases (78%) of which 16% were CA-MRSA [7]. In a study of impetigo in remote Aboriginal communities in northern Australia, *S. aureus* was isolated in 59% of impetigo lesions and 23% were CA-MRSA [8]. Furthermore, in northern Australia, the incidence of *S. aureus* bacteraemia in the Indigenous population was 5.8 times that of non-Indigenous Australians [9].

Infection due to *S. aureus* is thought to be the cause of significant disease in Pacific Island Countries (PICs) [10]. There is a large burden of impetigo due to *S. aureus* and *Streptococcus pyogenes* in many PICs, often due to secondary infection of scabies [11]. Although there are few population-based studies of *S. aureus* in the Pacific region, CA-MRSA is known to occur in the region. One particular strain of *S. aureus,* the so-called South-West Pacific (SWP) CA-MRSA clone, has been prevalent in in Samoan patients living in New Zealand, and also Pacific Island people populations in Australia [12]. Outbreaks of CA-MRSA have also been described in Pacific Island people populations in Hawaii [13], and amongst Samoans living in Alaska [14] and in Samoa itself [15].

Given the paucity of data regarding the clinical microbiological features as well as the molecular epidemiology of *S. aureus* from the Pacific Islands, the aim of this study was to describe the clinical and microbiologic epidemiology of infections amongst school children and hospital inpatients with *S. aureus* in the Republic of Fiji. Other aims included an examination of the incidence of *S.aureus* bacteraemia amongst different populations of Fijian residents. The study also sought to describe the frequency of MRSA infections among those with impetigo in schools to determine if a change was required from the current practice of using empiric beta lactam antibiotics for suspected *S.aureus* infections.

## Methods

### Setting

Fiji has a population of approximately 837,000 people living in some of its 330 islands in the tropical Western Pacific. The majority of the population is i-Taukei (Indigenous Fijian) with a large minority (37%) being Fijians of Indian descent [16]. We conducted two separate studies: the first was a prospective cohort survey of impetigo in 450 children over a period of 10 months in 3 primary schools in Fiji [11], and the second was a retrospective study of *S. aureus* isolated from clinical specimens sent to the microbiology laboratory at the Colonial War Memorial Hospital (CWMH) in the capital city of Suva.

### Community study

This was a prospective cohort study to investigate the part played by *S. aureus* in the causation of impetigo in school children aged 5 – 15 years in three schools conducted in 2006 [11]. Two of these were schools located in a rural area where the population was predominantly iTaukei, whilst the third (and largest) school was located in Suva with a population predominantly of Fijians of Indian descent. Each school was visited six times over a ten-month period at two monthly intervals and at each visit the children were examined for impetigo. Infected skin lesions were swabbed and swabs were placed in airtight bags with desiccant for transportation back to the laboratory in Suva. The swabs were plated onto sheep blood agar and incubated at 37°C for 24–48 hours [5]. *S. aureus* was identified by colony morphology and a latex slide agglutination test [Staphaurex Remel, Lenexa, KS, USA]. Isolates were stored in glycerol and transferred frozen to Darwin, Australia for further testing (described below).

### Hospital study

In this study isolates were collected from the diagnostic microbiology laboratory of the CWMH, Suva, the country’s largest hospital serving the Central Division of Fiji, which is inhabited by approximately one third of the nation’s population. Over a nine-month period September 2006 to May 2007, consecutive isolates of MRSA were collected from sterile and non-sterile sites and where possible, clinical and demographic data were collected from the patients’ medical record. During this time a small proportion (approximately 5% of the total identified in the laboratory) of MSSA isolates were collected (each MSSA isolate selected was identified temporally as close as possible to each MRSA isolate collected). This was done to ascertain some molecular epidemiological data of the MSSA isolates causing symptoms at the same time as the MRSA isolates that were being investigated. This yielded a similar number of MSSA as MRSA isolates, over the same time period. [From the four annual surveys, prior to this study, conducted by the scientist at the CWMH laboratory, approximately 85% of the *S.aureus* isolates came from swabs and 3-5% were MRSA (S. Pravin, personal communication)]. Also during this time the blood culture isolates of *S.aureus* were enumerated to calculate the incidence of *S.aureus* bacteraemia. We used the population of the Central Division of Fiji from the 2007 national census as the basis for denominator calculations. Because surveillance occurred during 2006 (4 months) and 2007 (5 months), we extrapolated a total population figure for 2006 using the pro rata difference between the last official census in 1996 and the latest census in 2007. In calculating average annualized rates, the denominator of person-months was calculated by multiplying each year’s population by the number of months of surveillance for each year (i.e. 2006 population by 4 months and 2007 population by 5 months) and adding these totals. The total number of cases over the 9 months was then divided by the total person-months and multiplied by 12 to give average annualized incidence rates with binomial exact 95% confidence intervals (CI). Incidence rate ratios were used to compare rates between ethnic groups.

### Antimicrobial susceptibility testing

Antimicrobial susceptibility testing was undertaken using the Vitek2 automated system [BioMerieux, Mary l’etoile, France] as per the manufacturer’s instructions. We considered non–multidrug-resistant MRSA (nmMRSA) isolates to represent CA-MRSA strains and considered multidrug-resistant MRSA (mMRSA) isolates to represent HA-MRSA strains [17]. nmMRSA isolates were defined phenotypically as those isolates resistant to beta-lactam antibiotics but resistant to no more than one other class of antibiotic, and mMRSA isolates were defined as those resistant to two or more classes of antibiotic in addition beta-lactam antibiotics. Antibiotic phenotype has previously been shown to accurately predict the genotype of CA-MRSA strains [18], and this result has been validated in various studies from Australia [6,9,17,19,20].

### Molecular typing

Using real-time polymerase chain reaction (PCR), *S. aureus* was verified by confirming the presence of *nucA* gene*,* MRSA was confirmed by the presence of *mecA* gene. The presence Panton Valentine leukocidin (PVL) has been associated with particularly virulent disease in some studies of CA-MRSA particularly. The ability to produce PVL was detected by the presence of *lukS*-PV-*lukF*-PV genes [21]. A proportion of the collected isolates were genotyped by means of a single-nucleotide polymorphism (SNP) genotyping system based on the multilocus sequence type (MLST) database, as previously elsewhere [8,21]. Of the 303 isolates 105 were selected for typing. These isolates came from throughout the whole collection period, and though not randomly chosen, there was no regard to individual isolate selection thereby avoiding bias as much as possible. In short, a kinetic PCR method was used to interrogate 8 highly discriminatory SNPs, allowing isolates to be assigned into eBURST [22] derived clonal complexes (CCs). Sequencing of selected MLST loci of some isolates was carried out using standard MLST primers and methodology for *S. aureus*[23]. Investigation of *spa* type diversity was performed using a high-resolution melting (HRM) assay of the *spa* locus [24].

### Statistical analysis

Data were analysed using non-parametric formulae in Stata version 9.1 (Stata-Corp, College Station, Texas, USA). The population-based incidence of *S. aureus* bacteraemia was calculated using the census data for the Central Division of Fiji as the denominator.

### Ethical approval

Ethical approval for this study was obtained from the Fiji National Research Ethics Review Committee, the Fiji National Health Research Committee and the Human Research Ethics Committee at the University of Melbourne. Children were only enrolled if written consent from a parent or guardian was obtained, and in the case of children aged ten years or older, if written assent was also obtained.

## Results

### Community study

A total of 563 swabs were taken from 455 children (108 children had two swabs taken at the same visit from two infected sites) and there was bacterial growth from 522 swabs (92.7%). There was growth of beta-haemolytic streptococci (predominantly *Streptococcus pyogenes*) from 491 swabs (87.2%), and *S. aureus* was isolated from 323 swabs (57.4%), co-existing with beta-haemolytic streptococci in 292 swabs (51.9%).

Antimicrobial resistance testing was performed on 299 of the 323 isolates. 20 isolates (6.7%, 95% CI 4.1 – 10.1) from 14 children were found to be nmMRSA, 17 underwent SNP-typing, and all were CC1 and PVL negative.

**Figure 1:**
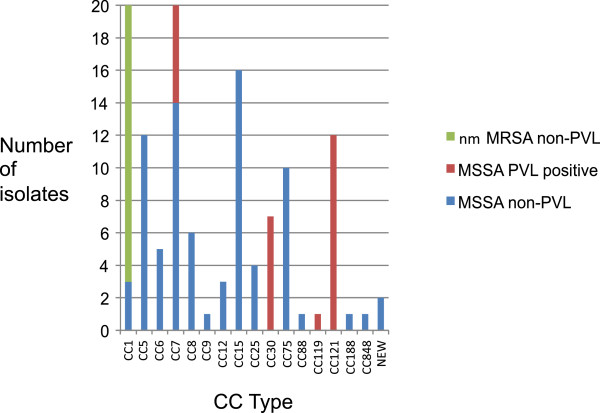
**Clonal complex types of *S.aureus *isolated from Fijian schoolchildren’s impetigo lesions.** Clonal complexes identified from SNP-typing of *Staphylococcus aureus* isolates collected from impetigo lesions in school children in Fiji (MSSA: methicillin sensitive *Staphylococcus aureus*; nmMRSA: non–multidrug-resistant methicillin resistant *Staphylococcus aureus*; PVL: Panton Valentine Leukocidin).

Of the remaining 303 MSSA isolates, 105 underwent SNP-typing. There was a wide distribution of CCs among the MSSA isolates (Figure 1) with the most common being CC5, 7, 14, 75 and 121. As the CC75 lineage has been noted to be non-pigmented in collections from northern Australia, we also demonstrated that all CC75 strains from this study were also non-pigmented when grown in LB broth. This CC75 lineage has provisionally been named *S. argenteus* given its divergent phylogenetic position [25] and non-pigmented phenotype and will herein be referred to as *S. argenteus*. Two isolates produced a previously undescribed SNP profile and full MLST revealed that they were a new MLST sequence type, which is a single locus variant of ST96. PVL positive strains comprised 26 of the 105 isolates and were all of the CC30 (7), CC119 (1) and CC121 (12) isolates and 6 of the 20 CC7 isolates.

### Hospital setting

**Figure 2:**
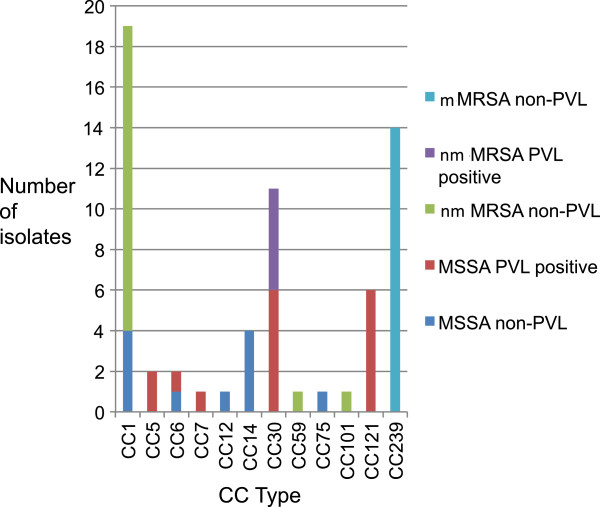
**Clonal complex types of *S.aureus *isolated from patients at CWMH, Fiji.** Clonal complexes identified from SNP typing of *Staphylococcus aureus* isolates collected from the Colonial War Memorial Hospital, Fiji (MSSA: methicillin sensitive *Staphylococcus aureus*; nmMRSA: non–multidrug-resistant methicillin resistant *Staphylococcus aureus*; mMRSA: multidrug-resistant MRSA; PVL: Panton Valentine Leukocidin).

Of the 36 MRSA isolates that underwent antimicrobial susceptibility testing and genotyping, 14 (39%) were mMRSA and all of these were of the globally recognised HA-MRSA lineage (ST239) and were PVL negative. The other 22 MRSA isolates were of the nmMRSA susceptibility phenotype, of which 5 (23%) were PVL positive (all were CC30). Of the remainder, fifteen were CC1, one was CC59 and one was CC101 (Figure 2).

Of the 27 MSSA isolates analysed, there were nine different CCs, and 59% (16/27) of isolates were PVL positive. To determine if a particular sub-lineage of the community-based CC1 strains was causing hospital infections we investigated diversity at the *spa* locus with HRM. We found that 3 HRM curves were present in similar proportions in both community and hospital isolates (community strains: HRM *spa* 1 (2 isolates), HRM *spa* 2 (14), HRM *spa* 3 (3); hospital strains: HRM *spa* 1 (1), HRM *spa* 2 (14), HRM *spa* 3 (3), non-typeable (2)).

#### Clinical features of patients with MRSA infection

Clinical information was available for 22 patients that had MRSA isolates. Eight of these 22 isolates were from infected surgical wounds, seven from non-surgical skin infections, two from CSF and one from blood culture. The median age was 43.5 years (range, 2 months–77 years); 55% were female; 16 were iTaukei, three were Fijians of Indian descent and 3 were ‘other’ ethnicity. Eight had had a history of hospitalisation or surgical procedures in the 12 months prior to the isolate being identified and 15 (68%) had received antibiotics (from CWMH) in the previous year. Eleven had diabetes mellitus and of these, five had MRSA isolated from diabetic foot infections. The median length of hospitalisation was 22 days (interquartile range, 15 to 36 days).

#### Incidence of S aureus bacteraemia

**Figure 3:**
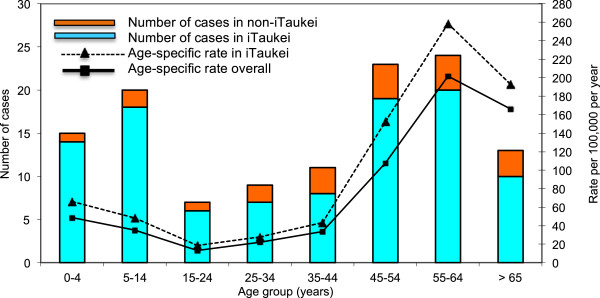
**Annualised incidence of *S.aureus *bacteraemia at CWMH, Fiji.** Number of cases and annualised incidence of cases where *Staphylococcus aureus* was cultured from blood at the Colonial War Memorial Hospital, Central Division, Fiji, May 2006 – September 2007 showing a predominance of cases amongst the iTaukei (Indigenous Fijian) population.

There were 128 separate episodes of *S. aureus* bacteraemia over the 9-month study period. This equated to an all-ages incidence figure for the Central Division of Fiji of *S. aureus* bacteraemia of 50.1 cases per 100,000 population (95% confidence interval, CI, 41.8 – 59.5) with peaks in children aged less than 5 years and adults aged 55 to 64 years (Figure 3). The incidence was highest in iTaukei (all-ages incidence 66.2 per 100,000, 95% CI 54.2 – 80.1), and iTaukei were three times as likely to have *S. aureus* bacteraemia compared with people of other ethnic backgrounds (incidence rate ratio 3.0, 95% CI 1.9 – 5.1). Only 3 of the 128 isolates were MRSA (2.3%). No further clinical information was available.

## Discussion

Although this study found a relatively low incidence of MRSA in Fiji in the community setting, the overall incidence of *S. aureus* bacteraemia was very high. There was considerable diversity of CC types seen amongst the CA-MRSA and the MSSA isolates, and this diversity was seen in both the community and hospital settings. While CC1 was the most common lineage observed and found both in the community and hospital studies, the CC239 clone was found to be well-established amongst hospital patients similar to more developed nations.

CC1 has also been found to represent 26% of MRSA strains in the Pacific Island nation of Samoa [15]. Further sub-typing of CC1 strains demonstrated that there was no over-representation of any particular community-based sub-lineage as a cause of hospital infections. CC239 is the typical HA-MRSA lineage found in Australia and Asia [26], and is evidently present in Fiji as a cause of nosocomial infections. With regards to CC30 MRSA, although the name SWP lineage would suggest an origin from this region of the world, it only comprised five of the 22 nmMRSA of hospital isolates and is most likely not a dominant lineage in either Fiji or Samoa [15].

Perhaps most surprising was the presence of *S. argenteus* strains. This divergent and early-branching lineage of *S. aureus* is prevalent in Indigenous populations in northern Australia, [8,27] and in an Amazonian tribe in French Guinea [28]. *S. argenteus* typically lacks the genes for production of the carotenoid pigment staphyloxanthin [25] and is hypothesised to be well adapted to causing skin infections and to be less virulent than other *S. aureus* lineages [29]. Findings from our current study extend the geographical distribution of this unusual lineage and confirm its non-pigmented phenotype. In addition, we provide supportive evidence regarding its virulence and epidemiological niche in that it represented 8.2% of community based impetigo isolates but only 1.6% of hospital based clinical isolates.

Our study found a prevalence of CA-MRSA in impetigo lesions among Fiji children of 6.7%, which is similar to a reported prevalence of 9% in Samoa [15]. This is in striking contrast to reports of high prevalence of CA-MRSA among Pacific Island peoples and Australian Aboriginal populations living in developed country settings [9,30,31]. The reason for the lower prevalence in Fiji is unclear, particularly as many of the conditions thought to contribute to the emergence of CA-MRSA in Indigenous populations in the developed world setting [6] are also present in Fiji. These factors are likely to include overcrowding, inadequate health-hardware for washing of clothes and people, poor skin hygiene and high rates of antibiotic use and further research specific to Fiji to investigate these is required. The prevalence of MRSA among hospital isolates of *S. aureus* in Fiji is also low; in the year prior to this study the proportion of all *S.aureus* isolates from the CWMH laboratory that were methicillin-resistant was 5% (personal communication, S. Pravin).

The incidence of SAB of 50.1 per 100,000 overall and 66.2 per 100,000 for the i-Taukei (Indigenous Fijian) population is considerably higher than that reported from developed countries [32] and is similar to that seen in Indigenous populations in both northern Australia (65 per 100,000) [9] and for the whole of Australia (62.5 per 100,000) [33]. The age distribution is also similar to that in Indigenous Australian populations with a peak incidence in the 45–65 age group. This is in stark contrast to that seen in developed countries, including the non-Indigenous population of Australia, where incidence increases with age [32,33]. Although we cannot infer a causative link, there is likely to be an association between high rates of skin and soft tissue infections and the high incidence of SAB in both Fiji and Indigenous Australian populations. Community-based efforts to improve skin health among these populations [34,35] will hopefully result in reduced rates of skin and soft tissue infections, and it will be important to determine if there is a concurrent reduction in incidence rates of community-onset SAB.

The hospital study identified consecutive patients that had MRSA isolated from clinical specimens sent to the hospital laboratory for culture, and in this small sample MRSA was primarily isolated from wounds (post-surgical or community acquired injuries). From the molecular typing data it appears that CC239 is commonly observed in hospital in Fiji and given the experience worldwide it would be expected that this is a nosocomial isolate. The nmMRSA CC1 isolates that were identified in the hospital setting however were most likely acquired in the community prior to admission, as the only MRSA detected in community study (in children that had likely not been to hospital) was CC1. However, these data come from a very brief snapshot of *S. aureus* infections at CWMH. It has been noted elsewhere that CA-MRSA isolates most frequently associated with community-associated infections can become established as the aetiology of nosocomial infections, [36] and therefore we cannot rule out this phenomenon occurring at CWMH.

Notably, of the hospital-based MSSA isolates, 59% carried *pvl* genes. While this represented only 16 of 27 isolates it is both surprising and concerning but is similar to findings in Africa [37] and Indigenous populations in northern Australia [29] where PVL was present in 57% and 40% of MSSA isolates respectively. Although we did not collect clinical information on the infections caused by these isolates, it nonetheless suggests that there is a large burden of PVL + MSSA disease given that MSSA represents >95% of *S. aureus* isolates at CWMH.

There are a number of limitations to our study. The community portion of our study investigated one type of clinical infection (impetigo) in one demographic group (school children) in two closely related regions of Fiji. A broader investigation of staphylococcal infections that includes older members of the community and a wider geographic region would be valuable. Within this school group there may also be may be an under-estimation of staphylococcal disease in this population as the surveys took place at two-monthly intervals it is likely that some episodes of impetigo occurred between visits and therefore were missed. The hospital portion of our study had a narrow time frame and a small numbers of cases (especially those where clinical information was available) and so the conclusions from this part of the study are limited.

## Conclusions

Despite these limitations it is clear that *S. aureus* is an important cause of disease in Fiji and there is considerable genotypic diversity in community skin infections in Fijian schoolchildren. CA- MRSA is present at a relatively low prevalence (6.7%) and in this survey was found to be due solely to CC1 (CA-MRSA). Isolates typically associated with both CA-MRSA and HA-MRSA isolates were found in the hospital setting with CC239 responsible for all of the HA-MRSA in this series. Beta-lactam agents, such as cloxacillin, remain the first choice for community-acquired *S. aureus* infections in Fiji. However, if the initial clinical response is poor then appropriate therapy for MRSA should be considered, such as clindamycin or vancomycin.

## Abbreviations

CC: Clonal complex; MRSA: Methicillin-resistant *S. aureus*; HA-MRSA: Healthcare-associated MRSA; CA-MRSA: Community-associated MRSA; PICs: Pacific island countries; SWP: South-West Pacific; CWMH: Colonial war memorial hospital; nmMRSA: Non–multidrug-resistant MRSA; mMRSA: Multidrug-resistant MRSA; PCR: Polymerase chain reaction; PVL: Panton-Valentine leukocidin; SNP: Single-nucleotide polymorphism; MLST: Multilocus sequence type; HRM: High-resolution melting.

## Competing interests

Authors declare that they have no competing interests.

## Authors’ contributions

AJ helped to devise the study and made substantial contributions to the acquisition of data, bacterial isolation and identification and writing the manuscript, DH carried out the molecular genetic studies, RR processed the specimens and isolated the bacteria in Fiji and assisted with molecular studies in Darwin, PS performed the antimicrobial susceptibility testing, SS supervised and assisted bacterial isolation, identification and susceptibility testing in Fiji, EB provided local Fiji data invaluable to the study design, JC significantly contributed to the study design and the drafting of manuscript, ST made major contribution to study design, analysis of data, and prepared most of the manuscript, AS devised the study, arranged the collection of samples, and gave significant assistance towards the drafting of manuscript. All authors read and approved the final manuscript.

## Pre-publication history

The pre-publication history for this paper can be accessed here:

http://www.biomedcentral.com/1471-2334/14/160/prepub
